# The role of alpha-fetoprotein in the tumor microenvironment of hepatocellular carcinoma

**DOI:** 10.3389/fonc.2024.1363695

**Published:** 2024-04-10

**Authors:** Yan Lu, Bo Lin, Mengsen Li

**Affiliations:** ^1^ Hainan Provincial Key Laboratory of Carcinogenesis and Intervention, Hainan Medical College, Haikou, Hainan, China; ^2^ Department of Medical Oncology, Second Affiliated Hospital, Hainan Medical College, Haikou, Hainan, China; ^3^ Institution of Tumor, Hainan Medical College, Haikou, Hainan, China

**Keywords:** hepatocellular carcinoma, AFP, tumor microenvironment, immune escape, liver cancer stem cells, immunotherapy

## Abstract

Hepatocellular carcinoma (HCC) is a prevalent malignant cancer worldwide, characterized by high morbidity and mortality rates. Alpha-fetoprotein (AFP) is a glycoprotein synthesized by the liver and yolk sac during fetal development. However, the serum levels of AFP exhibit a significant correlation with the onset and progression of HCC in adults. Extensive research has demonstrated that the tumor microenvironment (TME) plays a crucial role in the malignant transformation of HCC, and AFP is a key factor in the TME, promoting HCC development. The objective of this review was to analyze the existing knowledge regarding the role of AFP in the TME. Specifically, this review focused on the effect of AFP on various cells in the TME, tumor immune evasion, and clinical application of AFP in the diagnosis and treatment of HCC. These findings offer valuable insights into the clinical treatment of HCC.

## 1 Introduction

Hepatocellular carcinoma (HCC) is a prevalent malignant neoplasm worldwide, with the third highest fatality rate and increasing incidence and mortality rates (1–3). Despite substantial advancements in contemporary medical knowledge regarding the diagnosis and treatment of HCC, its incidence and mortality rates remain high. Given the high heterogeneity of HCC, early diagnosis and treatment play a crucial role in improving patients’ survival (4, 5). Alpha-fetoprotein (AFP) is an important tumor marker for diagnosing and treating liver cancer. It is also an important indicator for clinical diagnosis of liver cancer metastasis (6, 7). AFP, a glycoprotein primarily synthesized by the yolk sac and fetal liver during embryogenesis, is critically involved in sustaining embryonic development and placental functionality (8). However, the expression of AFP is extremely low in normal adults (9, 10). Nevertheless, when hepatocytes undergo malignant transformation, AFP expression exhibits a substantial increase (11, 12). Consequently, elevated AFP levels serve as a reliable indicator for the development and progression of HCC. Generally, high levels of AFP indicate the presence of HCC, and dynamic changes in AFP level can be used to predict the prognosis and response to treatment in HCC (7). However, there are also some patients with liver cancer who have normal AFP levels or only mildly elevated AFP levels, which may be related to differences in the type, location, and size of the tumor and the regulatory mechanism of AFP secretion (11).

The tumor microenvironment (TME) refers to the local tissue surrounding the tumor, including tumor cells, blood vessels, immune cells, stromal cells, and matrix molecules (13). The interaction between the TME and tumor cells plays an important role in tumorigenesis (14–16). HCCs caused by different etiologies (hepatitis B virus (HBV), hepatitis C virus (HCV), and chronic alcohol drinking) exhibit notable differences in their TME. Alcohol intake has inhibitory effects on immune cells in the TME, decreasing immune surveillance and enabling tumor cells to evade the immune response (17, 18). The expression level of AFP significantly increases during the progression of alcoholic liver disease to HCC (19). However, the molecular mechanisms underlying this process are still poorly understood. Chronic infections with HBV and HCV can lead to immune tolerance, allowing the viral infection to persist and cause hepatocyte damage, finally leading to carcinogenesis (20, 21). Studies have found that in the TME, the X protein of HBV can stimulate the expression of reprogramming-related proteins by increasing AFP expression, thus inducing the proliferation of liver cancer stem cells (LCSCs) (22). HCV infection, on the other hand, upregulates AFP expression, which decreases the activity of NK cells and reduces CD4^+^ cells in the TME (23, 24). The immune dysregulation in the TME of HCV-related HCC patients is closely related to the elevated levels of AFP. Although there are significant differences in the TME, the function of AFP in the TME remains consistent. AFP promotes the formation of an immune-tolerant microenvironment and facilitates immune evasion of tumor cells.

As a marker of HCC, AFP plays an important regulatory role in the TME. However, the current role of AFP in the TME of HCC is not fully understood. Therefore, a deeper insight into the role of AFP in the TME is of great significance for the early diagnosis and treatment of HCC. This article summarizes the role of AFP in the TME of HCC, especially discussing its effect on liver cancer stem cells, tumor-associated macrophages, cancer-associated fibroblasts (CAFs), endothelial cells, mesenchymal stem cells, tumor immune escape, etc., providing a reference for the clinical diagnosis and treatment of HCC.

## 2 The basic structure and function of AFP

**Figure 1:**
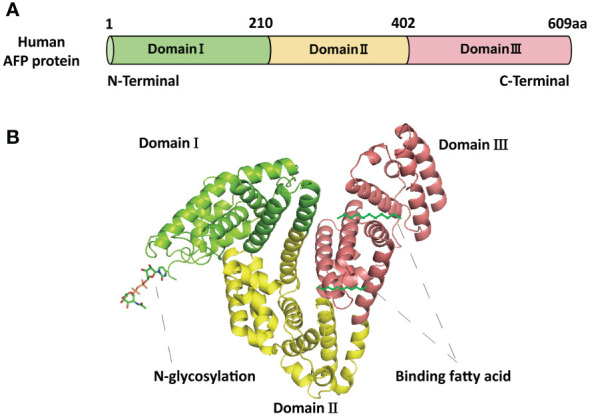
The basic structure of AFP. **(A)** The schematic diagram of the human AFP protein sequence with three domains. **(B)** The structure of AFP and the binding sites of AFP with fatty acids and N-glycosylation. The structure of AFP displays a V-shaped configuration, consisting of domain I (N-terminal) on the left side of the V, domain III (C-terminal) on the right side of the V, and domain II (middle region) at the base of the V.

AFP is a fetal-specific alpha-globulin synthesized during fetal development and present in fetal blood and tissues. It can also be detected in adult liver and some malignant tumors (25). Human AFP is similar to albumin, composed of a single polypeptide chain with a molecular weight of nearly 69 kDa (25, 26). The single-chain polypeptide of AFP consists of 609 amino acid residues. These amino acid residues are arranged in a specific order to form three structural domains, namely the N-terminal domain (1-210 amino acid residues; domain I), the central domain (211-402 amino acid residues; domain II), and the C-terminal domain (403-609; domain III) (27) (**Figure 1A**). These three domains of AFP are linked by disulfide bonds to form a V-shaped structure (**Figure 1B**). AFP possesses three distinct structural domains, each exhibiting diverse biological activities. The N-terminal domain can interact with other molecules, thereby affecting the function of AFP. For example, previous studies have found that AFP domain I can bind to the phosphatase domain of PTEN, thereby affecting PTEN activity (25). The central domain (domain II) consists of approximately 192 amino acid residues, possesses a high degree of flexibility, and is easily digested by proteases. The C-terminal domain (domain III) is the last domain of AFP, which is the most conserved domain, consisting of about 207 amino acid residues. This domain possesses a continuous sequence of several hydrophobic amino acids forming a leucine zipper-like structure. Domain III is responsible for binding to signaling proteins and receptors, thereby regulating their biological activity (26–28). Studies also found that AFP domain III can bind to the C2 domain of PTEN, forming the AFP-PTEN complex, inhibiting PTEN, activating the PI3K/AKT signaling pathway, and promoting HCC progression (25). Domain III can also bind to mucin, scavenger receptors, chemokines, etc. After binding to its receptor, AFP is endocytosed and packaged with the receptor and transported to the cell organelles via the Golgi complex, where it is degraded or activates/blocks cell signaling pathways (26–28).

AFP secretion and expression are not consistent with HCC development. One reason is that AFP has two basic forms: native AFP (nAFP) and tumor-derived AFP (tAFP). nAFP is mainly produced by the fetal yolk sac and fetal liver and secreted into fetal circulation. It is a normal plasma protein and does not promote HCC development. Sometimes, adult individuals express nAFP, which is mainly observed during physiological cell regeneration and hematopoiesis. However, tAFP expressed in TME mainly originates from HCC and can support tumor growth and metastasis. Interestingly, tAFP is not a mutated form of nAFP and differs only slightly in terms of glycosylation and lipid-binding characteristics (29, 30).

AFP described in this article specifically refers to tAFP. AFP exhibits numerous variability, thereby presenting considerable challenges in comprehending its biological activity with HCC (31). AFP variability relies on the types of species, tissues, isoforms, binding ligands, binding partners, and post-translational modifications such as N-glycosylation (**Figure 1B**). Different variants of AFP possess different biological functions (29); for example, it was found that the serum levels of fucosylated AFP variant increased in patients with HCC but were undetectable in the serum of normal patients (32). Polyunsaturated fatty acids binding AFP variants play an important role in the TME and affect dendritic cells (DCs) and natural killer cells (NK cells) activation (33). Despite extensive studies on the structure of AFP, its biological functions remain unclear. Further studies are imperative to unveil its precise association with various physiological and pathological processes.

## 3 The role of AFP in the TME of HCC

The interplay among cells within the TME is a crucial factor in tumor progression, metastasis, and treatment (34, 35). AFP, as a tumor biomarker, directly modulates tumor cell behavior and the TME by interacting with tumor-associated cells and molecules.

### 3.1 AFP is closely related to liver cancer stem cells

Liver cancer stem cells (LCSCs), also known as liver cancer-initiating cells, refer to a subpopulation of cells with self-renewal capacity and stem cell characteristics in the HCC microenvironment. They can produce heterogeneous tumors, are highly invasive and tumorigenic, and can drive tumor growth, metastasis, and recurrence (12, 36). Targeted killing of LCSCs can ameliorate tumor drug resistance and recurrence while preserving normal tissue activity with high specificity. Due to the presence of various subpopulations of tumor cells expressing different markers in HCC, some scholars believe that LCSCs are the key to the development and heterogeneity of HCC (37, 38).

**Figure 2:**
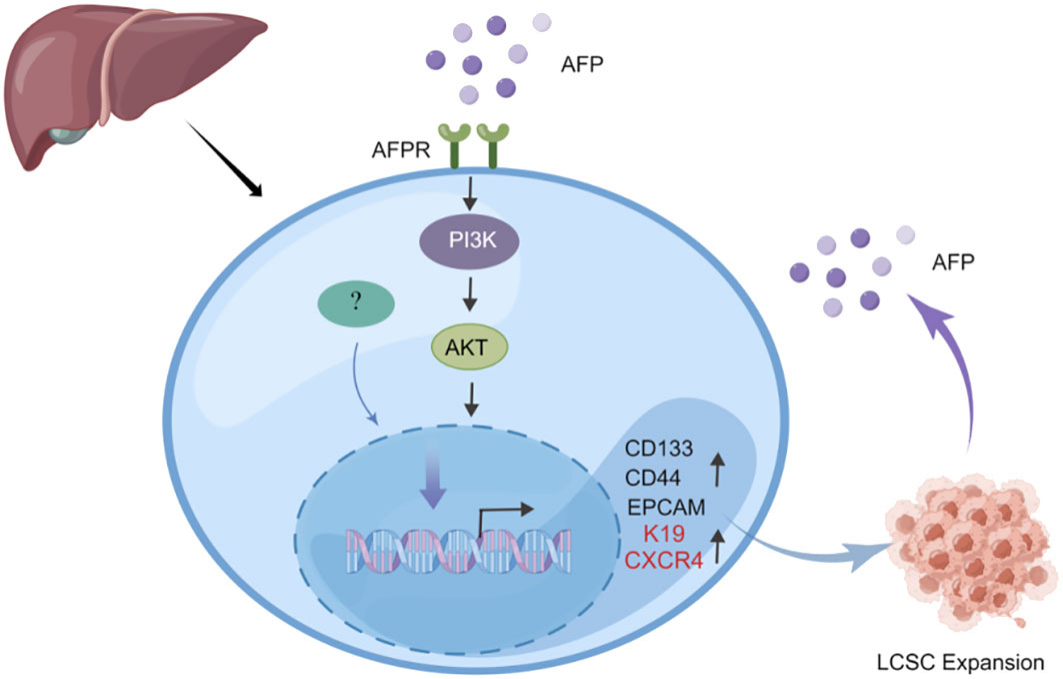
AFP can promote the expansion of LCSCs. AFP upregulates the expression of CD133/CD44 and EPCAM by activating the PI3K/AKT signaling pathway, thereby promoting the expansion of LCSCs. In addition, AFP can directly upregulate the expression of LCSCs marker proteins K19 and CXCR4 to accelerate liver cancer progression.

Previous studies indicated that AFP can enhance the expansion of LCSCs by activating the PI3K/Akt signaling pathway (39, 40). To investigate the impact of AFP on LCSCs, researchers measured the AFP expression in HCC cells firstly, then demonstrated that AFP not only facilitates the expression of proteins associated with cell reprogramming but also promotes the expression of LCSCs markers, including CD44, CD133, and EpCAM. Further analyses revealed that AFP can induce the malignant transformation of liver cells by activating the PI3K/AKT signaling pathway, thereby stimulating the expression of reprogramming-related proteins and oncogenes and inducing the generation of stem cells (39) (**Figure 2**). Simultaneously, several studies have demonstrated that AFP can accelerate liver cancer progression by upregulating LCSC markers K19 and CXCR4 (**Figure 2**) (41–44). It has also been suggested that AFP-induced tumor stem cells can secrete AFP (43). LCSCs-derived AFP can facilitate immune evasion by regulating the behavior of crucial immune cells in liver cancer cells. Consequently, it has been suggested that AFP can serve as a biomarker for LCSCs (44). AFP primarily facilitates stem cell expansion and preserves stemness. These effects enhance the survival and metastatic potential of LCSCs, thereby fostering the initiation and progression of HCC. Subsequent investigations should elucidate the precise mechanism underlying the interaction between AFP and LCSCs and explore intervention approaches targeting AFP to enhance the treatment effect of liver cancer.

### 3.2 AFP can inhibit the phagocytic ability of HCC-associated macrophages

A significant number of macrophages infiltrate the tumor stroma, referred to as tumor-associated macrophages (TAMs) (45). Experimental evidence indicated that TAMs possess immune regulatory functions and are closely associated with tumor growth and progression (46–48). Tumor cell-derived chemokines, such as CCL2-8 and VEGF, can attract macrophages into the TME and facilitate their differentiation into the M2 phenotype, thereby promoting tumor progression (49). TAMs play a crucial role in the malignant progression of HCC (50–52).

Previous studies have demonstrated that purified human recombinant AFP protein can prevent the phagocytosis of chicken red blood cells by macrophages. Downregulation of AFP dampens this inhibitory effect. Subsequent studies have elucidated that AFP can impede the phagocytic activity of macrophages toward hepatoma cells and other tumor cells through its interaction with macrophage receptors (53–55). A recent study demonstrated that AFP can affect the function of macrophages in phagocytizing liver cancer cells by inducing the polarization of TAMs (55). This study employed human monocytic leukemia cells (THP-1) and monocytes from healthy donors to measure the effect of AFP on macrophage phenotype and phagocytosis. Their findings revealed that AFP can facilitate the polarization of macrophages toward the M2 phenotype and undermine the phagocytic ability of M1 macrophages toward liver cancer cells. Further analyses revealed that this process is related to the activation of the PI3K/Akt signaling pathway. Therefore, AFP is a key cytokine that inhibits liver cancer cell phagocytosis by macrophages (54).

The effect of AFP on TAMs is multifaceted. It is involved in the initiation and progression of HCC, thus holding significant implications for the design of immunotherapeutic approaches for HCC. Nevertheless, research into the influence of AFP on TAMs remains nascent. Further elucidation of the mechanisms involved in the interaction between AFP and TAMs and potential intervention strategies can undoubtedly enhance the clinical efficacy of immunotherapy for liver cancer.

### 3.3 AFP regulates the immune escape of liver cancer cells

Immune surveillance serves as the principal protective mechanism of the human body against external intrusion or internal genetic alteration, enabling the identification and eradication of tumor cells. Nevertheless, mutant cells can evade immune surveillance through diverse mechanisms, accelerating their proliferation and metastasis, a phenomenon commonly referred to as tumor immune escape. The mechanisms underlying immune evasion of tumor cells encompass a typical expression of antigens on the tumor cell surface, modification of the molecular structure of the tumor cell surface, and secretion of immunosuppressive factors (56).

Based on recent findings, AFP can inhibit the activity of various immune cells, such as DCs, NK cells, and T cells (56, 57). Specifically, AFP can reduce the antigen presentation ability of DCs, thereby preventing DC-mediated activation of T cells (58, 59). It was found that plasmacytoid DCs (pDCs, immunosuppressive DCs) are abundant and localized in type 1 Tregs and promote the production of IL-10 in the TME of HCC. AFP levels correlated with the high numbers of pDCs, tumor metastasis, and increased tumor infiltration of Tregs (2).Studies have confirmed that AFP isolated from human umbilical cord blood acts on monocytes in patients with liver cancer, inhibiting the conversion of monocytes into mature DCs. Since AFP can inhibit the maturation of DCs, it can promote tumor immune escape (29, 60).

**Figure 3:**
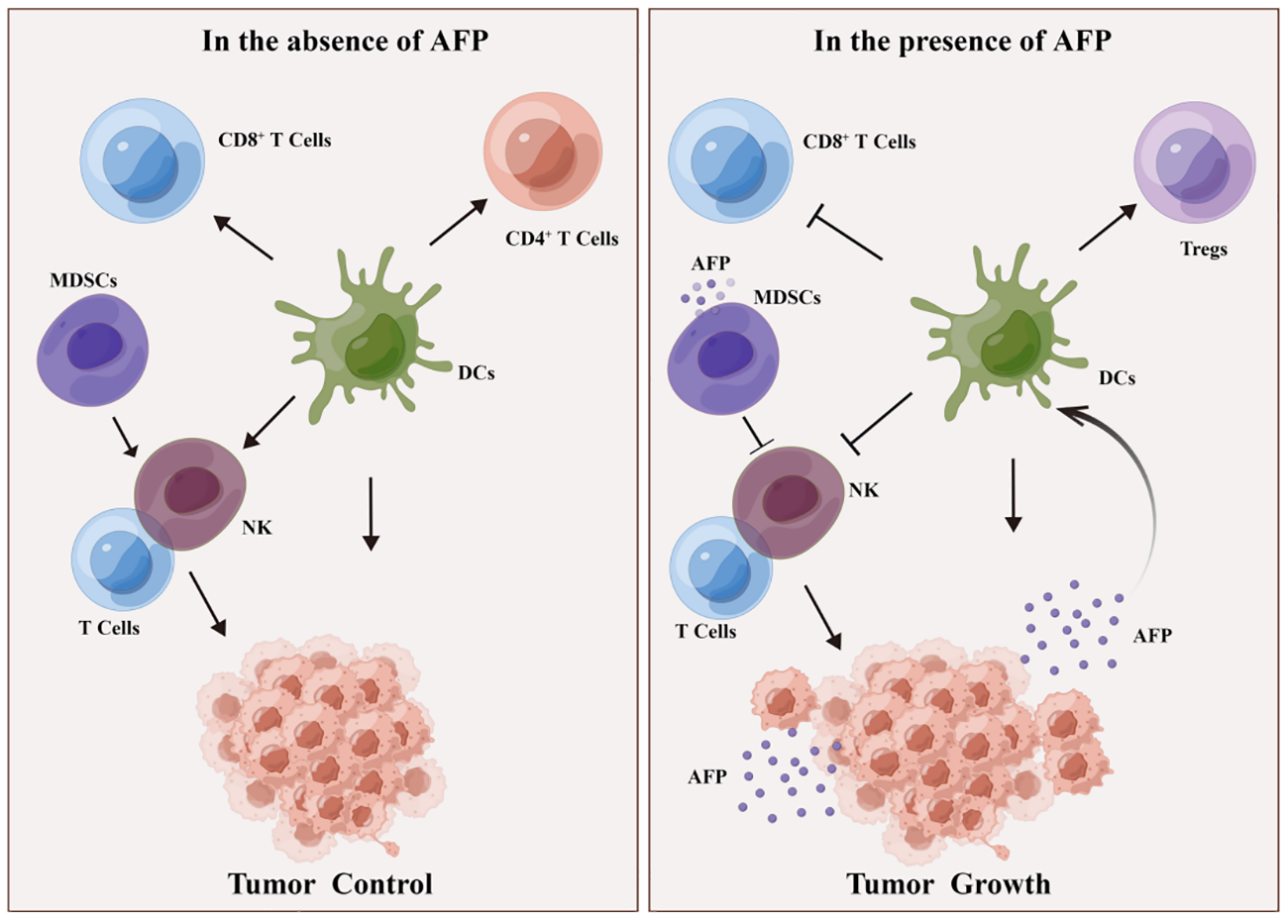
DCs and MDSCs involved in AFP-induced immune escape. In the absence of AFP, DCs enhance the function of CD4+ T cells, CD8+ T cells, and NK lymphocytes, thereby impeding tumor growth. MDSCs activate T and NK lymphocytes, inhibiting tumorigenesis. In the presence of AFP, DCs promote Treg differentiation but suppress the function of CD8+ T cells and NK cells, consequently fostering an immunosuppressive milieu and facilitating tumor growth. Additionally, in the presence of AFP, MDSCs hinder the function of T cells and NK cells, thereby promoting tumorigenesis.

Simultaneously, AFP can attenuate its cytotoxicity toward liver cancer cells by regulating signaling pathways in NK cells (54). AFP typically does not directly impair the function of NK cells; instead, it indirectly hampers their function by impeding the maturation of DCs and decreasing IL-12 secretion by DCs (54, 56). Furthermore, AFP also suppresses the proliferation and cytotoxicity of T cells, thereby attenuating their response to liver cancer cells. AFP can hinder the immune response by inducing lymphocyte apoptosis and modulating T lymphocyte differentiation into CD4^+^ T cells and CD8^+^ T cells. AFP promotes DC-mediated differentiation of regulatory T cells (Tregs) and dampens the function of CD8^+^T cells and NK cells, thus inducing an immunosuppressive environment and tumor progression (**Figure 3**). Despite the limited immunogenicity of AFP, it can provoke immune evasion by suppressing the function of DCs, NK cells, and T lymphocytes, forming an immunosuppressive microenvironment, thereby promoting tumor progression. Myeloid-derived suppressor cells (MDSCs) are a subset of immature myeloid cells originating from myeloid hematopoietic stem cells. These cells express CD11b, CD14, CD15, and CD33 antigens but do not differentiate into macrophages, DCs, or granulocytes. MDSCs possess AFP receptors on their cell membranes, which interact with AFP and inhibit the function of NK cells and T lymphocytes. Consequently, this interaction establishes a microenvironment conducive to immune evasion in cancer (30) (**Figure 3**).

AFP diversely affects the immune evasion of HCC; for example, it inhibits immune cell activity, disrupts antigen presentation, and induces immune tolerance. These effects collectively enable liver cancer cells to evade immune surveillance, thereby augmenting their immune evasion. Therefore, investigating the mechanisms governing the interaction between AFP and immune evasion of liver cancer holds promise for the discovery of novel approaches for immunotherapy in HCC.

### 3.4 AFP may interact with cancer-associated fibroblasts (CAFs) and affect HCC tumor growth and invasion

Cancer-associated fibroblasts (CAFs) are the most important cell population in the TME. They promote HCC growth and drug resistance. They mediate HCC progression by activating signaling pathways, such as the JAK/STAT, MAPK, and Wnt/β-catenin pathways (61). AFP also activates these signaling pathways to promote the malignant transformation of cancer cells. The interaction of AFP with CAFs is unclear, but AFP can promote HCC development in patients with liver fibrosis. The blood levels of secreted AFP can be used to accurately stratify patients with advanced liver fibrosis for their HCC risk and guide HCC screening (62). Eliminating serum lectin-reactive alpha-fetoprotein can suppress post-treatment recurrence of HCC in cirrhotic patients (63).

### 3.5 AFP induces endothelial cells to promote endothelial-to-mesenchymal transition

The liver is formed by hexagonally shaped anatomical units named ‘liver lobules’. Blood flows through sinusoidal channels of the liver lobules and drained into central veins. Sinusoidal endothelial cells form the wall of liver sinusoids which play critical roles in liver homeostasis (64). Liver Sinusoidal endothelial cells have multiple fenestrate that facilitate transferring substrates between blood and the extravascular compartment (65).

Sinusoidal endothelial cells play important roles in cancer development. In TME, sinusoidal endothelial cells become capillarized or defenestrated after exposure to inflammatory factors, such as IL-6 and AFP, which promote liver fibrosis, cirrhosis, and liver cancer. AFP is expressed around fibrotic bridges in chronic liver damage and at the margins of necrotic tissues. It promotes the recruitment and proliferation of endothelial cells, hepatic stellate cells (HSCs), and macrophages. This may imply AFP reprograms live cells and remodels the extracellular matrix to promote endothelial-to-mesenchymal transition and tumor angiogenesis (66, 67). It was found that ramucirumab, a monoclonal antibody specific for VEGFR2, combined with tyrosine kinase inhibitors regorafenib and cabozantinib, present specific benefits for advanced HCC patients with high serum concentrations of AFP (68).

### 3.6 AFP mediates mesenchymal stem cells

Mesenchymal stem cells (MSCs) are multipotent cells initially discovered from bone marrow. MSCs can be differentiated into chondrocytes, osteocytes, adipocytes, myocytes, astrocytes, and other cells *in vitro*. MSCs can be recruited into the TME and promote or inhibit the development of HCC. The contradictory effects of MSCs mainly come from TME of HCC, and whether these cells are endogenous or exogenous. In TME, endogenous MSCs can promote hepatic fibrosis by regulating the inflammatory microenvironment. However, in clinical trials, exogenous MSCs were used to treat hepatic fibrosis (69, 70).

It was found that exogenous MSCs can suppress tumor growth in animal experiments. For example, co-cultured umbilical cord-derived MSCs with hepatocarcinoma cell line HepG2 downregulated the protein expression of AFP, Bcl-2, and survivin and accelerated cancer cell apoptosis, which was related to the apoptosis signal pathway. Thus, exogenous umbilical cord-derived MSCs can inhibit growth and promote the apoptosis of cancer cells by downregulating AFP, Bcl-2, and survivin (71).

Exogenous MSCs are a potential source of stem cells for cell therapy in treating liver cirrhosis due to their many advantages. MSCs are easy to obtain and easy to culture without losing their characteristics. Exogenous MSCs can be recruited into the injury sites and TME and have strong immunoregulatory abilities. In addition, they have multiple differentiation potential and capacity to repair injuries and regenerate (70). Several clinical trials have investigated the potential of MSCs in treating liver cirrhosis, particularly alcoholic liver cirrhosis (NCT02705742, NCT03626090, NCT05080465, NCT05227846, and NCT05155657). Satisfactory outcomes have been observed in clinical trials of exogenous MSCs. New strategies are needed to improve cell sources for recipients. In the future, synergistic downregulation of AFP with exogenous MSCs may improve their therapeutic efficacy in liver cirrhosis and HCC (70).

## 4 The role of AFP in the diagnosis and treatment of HCC

AFP is as a serological biomarker for HCC screening, diagnosis, treatment response monitoring, and prognosis. The sensitivity and specificity of AFP for diagnosing HCC are 41–65% and 80–94%, respectively. However, nearly 50% of patients with HCC are AFP-negative and AFP may be elevated in benign liver diseases, such as hepatitis and cirrhosis (72, 73).

AFP-negative and AFP-positive patients display differential proteomic profiles and metabolomic profiles. The symptoms of AFP-negative patients are generally mild and they benefit more from treatment compared with AFP-positive patients. AFP level has strong relationships with malignant features of HCC (72, 73). The analysis of tumor-infiltrating lymphocytes in AFP-positive TME reveals an enrichment of Tregs and exhausted CD8^+^ T cells. Accordingly, the number of cytotoxic T-lymphocyte associated protein 4 (CTLA4) and programmed death 1 (PD-1) T cells is higher in AFP-positive patients than in AFP-negative patients. Also, high abundances of regulatory T cells were found in AFP-positive patients, which drive cytotoxic T cell dysfunction in the TME (74).

TAMs, DCs, and monocytes were more abundant in the adjacent normal tissues of AFP-positive patients. AFP upregulates IL-6, transforming growth factor beta 1 (TGF-β1), CXCR4 and NF-κB, to recruit TAMs into TME. TAMs promote the expression of angiogenesis-associated genes and downregulate phagocytosis and lymphocyte response, which may recruit fibroblasts and induce extracellular matrix remodeling to promote tumorigenesis (75).

Elevated serum levels of AFP typically signify the existence or recurrence of HCC, thus rendering AFP a valuable tool for diagnosis and treatment (76).Some patients with HCC are AFP-negative preoperatively; however, AFP may increase after surgery. AFP can acutely elevate for about 5 days after partial hepatectomy (77). Rising concentrations of AFP are closely associated with hepatocyte regeneration. AFP can be synthesized and released by hepatocytes undergoing proliferation and mitosis after operation (78). AFP expression was also seen in the regenerative phase after liver injury, which was associated with hepatocyte proliferation. During proliferation, AFP is produced by hepatocytes and released into the microenvironment and circulation. It was also found that AFP is not only related to cell proliferation but also related to cellular death and apoptosis (52). After surgery, AFP level will decline with healing. However, in postoperative recurrence, AFP is reproduced by HCC cells and cancer stem cells. AFP production during postoperative recurrence is related to fast-growing, poorly differentiated, and malignant HCC cells (44). HCC is an extraordinarily heterogeneous malignant liver cancer. Preoperative and postoperative recurrence of HCC are different in molecular characteristics, signal transduction, and genomic instability. They are characterized by great heterogeneity. AFP can arise from cancer stem cells, which are silent in patients before surgery. These cells are more active in the TME during hepatocyte necrosis, myeloid cell migration, immune evasion, and matrix remodeling after postoperative recurrence (44, 79, 80). Thus, AFP not only functions as a marker but also aids in the interpretation of imaging in liver cancer. The value of AFP can be associated with the dimensions, progression, and prognosis of HCC. Elevated levels of AFP may serve as an indicator of a larger or more aggressive tumor and an unfavorable prognosis (81). Moreover, post-treatment monitoring of AFP levels in patients with liver cancer can be employed to assess treatment efficacy and disease recurrence. A substantial decrease in AFP level following treatment generally signifies successful intervention, whereas an increase in AFP level may suggest tumor relapse or progression (60). Thus, AFP plays a crucial role in the diagnosis and management of HCC. As a marker, it can be used for early screening, diagnosis, and follow-up of patients with liver cancer. In addition, AFP levels can also be used to evaluate the therapeutic effect and predict the prognosis.

Recently, AFP has increasingly gained interest in HCC immunotherapy. AFP can be applied in immunization. Particularly, it can be a marker for immunotherapy and predict the efficacy of immunotherapy. AFP is highly expressed in nearly 65% of patients with HCC, and serum AFP is as a biomarker for diagnosing and treating HCC because it is inversely correlated with prognosis (2, 82). HCC patients cannot tolerate to high serum levels of AFP which induce significant tumor burdens. AFP is a tumor-associated antigen, and cytotoxic T lymphocyte (CTL) epitopes for AFP were identified during tumorigenesis (83, 84). Patients with high circulating levels of AFP have strong T cell responses. Both murine and human T cells can recognize the self-antigen AFP, indicating that it can serve as a tumor rejection antigen. AFP-based CAR-T cells and AFP-based vaccines have shown an antitumor immune response *in vivo* and in clinical trials (85, 86).

AFP is produced intracellularly and secreted by HCC, making it “untargetable” for chimeric antigen receptor T (CAR-T) cell immunotherapy. However, peptides derived from AFP are processed and presented by class I MHC on the surface of HCC cells. Therefore, antibodies against AFP-derived peptides/MHC complexes can be designed and engineered into CAR-T cells. CAR-T cell therapy was successful in liver cancer xenograft models *in vivo* (85). Also, CD8+ T cells were found in transgenic mice that recognized AFP-derived peptide epitope on human HCC cells. Adoptive transfer of AFP-derived peptide-specific CD8^+^ T cells eradicated HepG2 tumor xenografts in mice (87).

Preclinical studies found that AFP-derived peptide-specific TCR-engineered T cells are unlikely to cause severe off-target toxicity (88). AFP peptide-based CAR-T therapy in advanced HCC is being tested in a phase I clinical trial (NCT03132792) to find the safety of adoptive transfer of engineered T cells, which target AFP-positive liver cancer (89).

Dysfunction of DCs is also the main mechanism of tumor immune escape. Vaccination can promote the activity of DCs and stimulate antitumor responses in HCC. The beneficial immune responses of CD8^+^ and CD4^+^ T cells can be generated if AFP is presented by more AFP-engineered DCs. It was found that AFP-pulsed DCs can shift specific cytotoxic T lymphocytes toward AFP-producing HCC cells (90, 91).

Using AFP-coding adenoviruses to transduce DCs can induce the antitumor immune response, delay tumor growth, and improve long-term survival *in vivo* subcutaneous HCCs. The combination of the AFP-DCs vaccine with CD40 ligand-DCs vaccine has shown synergistic results. It changes the TME, enhances the tumor infiltration of cytotoxic T lymphocytes and DCs, and upregulates Th1-derived cytokines, leading to tumor apoptosis and regression (92, 93). A phase I/II trial measuring immunization in HCC patients found that AFP-DCs vaccine can produce AFP-specific T cell responses with increasing IFN-γ in the environment of high oncofetal antigen (86). Nevertheless, more comprehensive studies are imperative to ascertain the efficacy and safety of these approaches and vaccines before their implementation in clinical settings.

## 5 Other therapeutic strategies and challenges of AFP in the TME

TME is a complex ecosystem comprising tumor cells, blood vessels, immune cells, stromal cells, and matrix molecules. AFP and its downstream pathways play a key role in this system, making them targets for novel tumor treatment. The expression level of AFP is associated with tumorigenesis and tumor prognosis (94, 95). Hence, therapeutic strategies that target AFP or its downstream pathways may be beneficial for treating these tumors. One potential therapeutic approach is to inhibit the expression or activity of AFP. For instance, using small interfering RNA (siRNA) or antisense oligonucleotides (ASO) technology to reduce AFP expression. These technologies can specifically inhibit the synthesis of AFP or prevent its interaction with target cells, thereby suppressing tumor growth and diffusion (96, 97). Secondly, the use of recombinant AFP, combined with anticancer drugs, has been widely explored. Besides, recombinant AFP fragment derived from AFP domain-3 was found to suppress tumor cell growth. Various anticancer drugs exhibited therapeutic prospects when combined with AFP or AFP fragments, such as carminomycin, doxorubicin, paclitaxel, sorafenib, and maytansinoid (98–103). Moreover, AFP can activate downstream signaling pathways, such as PI3K/Akt in tumor cells, promoting proliferation, survival, and migration (22, 104). Targeting these pathways with small-molecule inhibitors or antibodies can inhibit tumor growth. The complexity of TME makes a single therapy ineffective. Combining multiple strategies, like targeting AFP and its downstream pathways with immunotherapy and radiotherapy, may more effectively control tumor growth and invasion.

In the TME, AFP-targeted therapies still face challenges and limitations. Firstly, the expression level of AFP has limited sensitivity and specificity for HCC patients, it is not a marker for all HCC, and about one-third of the patients with advanced HCC have no AFP expression (105, 106). Moreover, patients with acute and chronic liver without evidence of HCC may have high AFP elevation. Therefore, therapeutic strategies targeting AFP may not be applicable to all HCC patients. Secondly, the heterogeneity of the tumor microenvironment is another challenge. AFP expression varies among different types and stages of HCC (7, 105), and the accuracy value with advanced HCC varies according to patient characteristics (7). Due to the significant differences in cell types, gene expression, and signal pathway activation among different regions of a tumor (106). Drug resistance is another concern. Tumor cells may adapt to treatment pressure by activating other signaling pathways or mutations, leading to treatment failure (107). Overall, there are challenges in AFP-targeted therapies, and future studies are needed to understand the mechanism by which AFP is involved in tumorigenesis and assess the efficacy and safety of AFP-based treatment strategies.

AFP utility is limited and challenges, however, targeting the AFP-TME interaction in HCC treatment has significant benefits to AFP positive patients (75). Blocking the function of AFP or inhibiting its interaction with TME can disrupt the connection between liver cancer cells and TME, thereby inhibiting tumor growth and diffusion. This strategy may provide a new approach for treating HCC. Secondly, AFP is also involved in the interaction between liver cancer cells and the immune microenvironment (29). AFP overexpression can promote the immune evasion of HCC cells, protecting them from the immune response. Targeting AFP may help attenuate this immune evasion, enhancing the immune recognition and response, thereby improving the efficacy of immunotherapy. In summary, targeting the AFP-TME interaction in HCC can disrupt the link between liver cancer cells and the TME, inhibiting tumor growth, invasion, and immune evasion.

## 6 Conclusion

**Figure 4:**
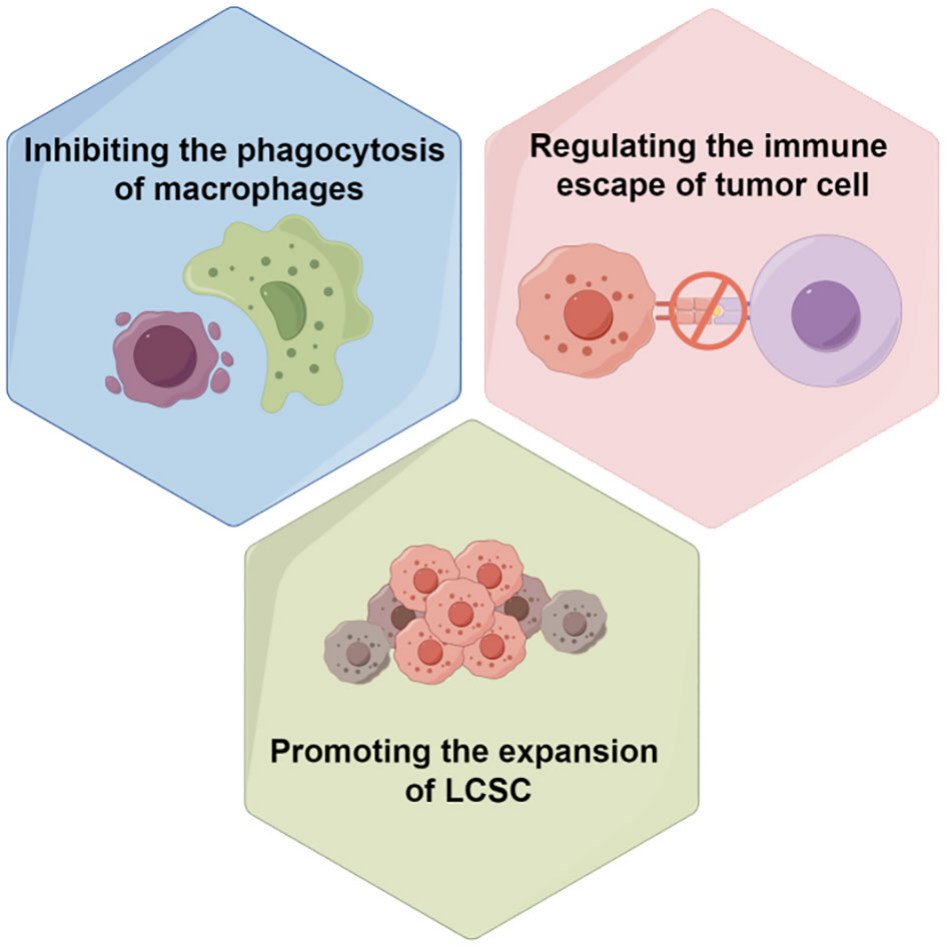
The role of AFP in the TME of HCC.

**Figure 5:**
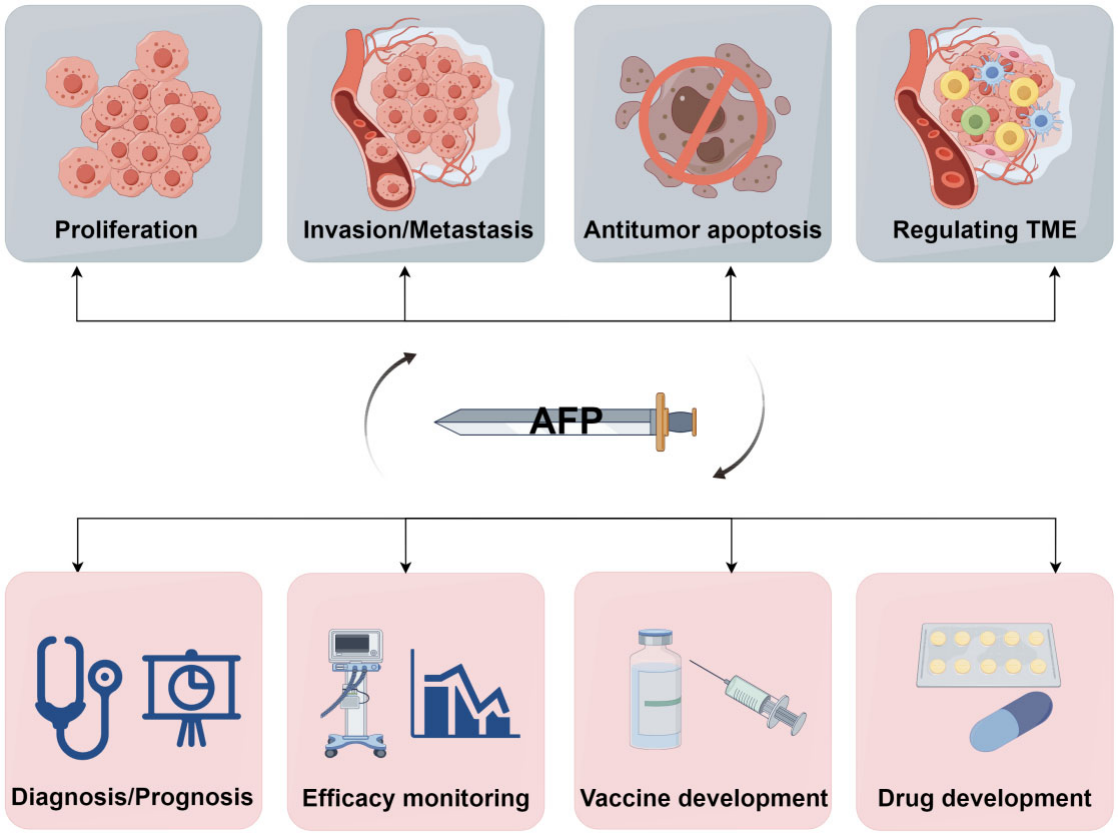
A double-edged sword of AFP. AFP is a double-edged sword due to its role as both an oncofetal antigen and a diagnostic marker for HCC. Its participation in various aspects of tumor biology, including proliferation, invasion, metastasis, apoptosis, and TME regulation, underscores its potential detrimental effects. Conversely, measuring AFP levels can help early diagnosis, prognostic evaluation, monitoring of treatment efficacy, and advancement of therapeutic interventions in HCC. Additionally, AFP can serve as a valuable target for developing drugs and vaccines (by Figdraw 2.0).

In conclusion, AFP holds significant value as an HCC biomarker, and its high expression is intricately linked to the development and progression of HCC. AFP can stimulate the expansion of LCSCs in the TME by activating the PI3K/Akt signaling pathway. Additionally, AFP can facilitate HCC progression by upregulating the expression of genes associated with LCSCs. Furthermore, AFP can undermine macrophage-mediated phagocytosis of tumor cells by interacting with macrophage receptors and inducing tumor immune escape. Simultaneously, AFP may also suppress the function of NK cells, macrophages, DCs, CAFs, endothelial cells, and mesenchymal stem cells, and impair the ability of cytotoxic T lymphocytes to eliminate tumor cells, thus facilitating tumor immune evasion (**Figure 4**). Recent findings have demonstrated that AFP inhibition can effectively inhibit the malignant behaviors of HCC cells, suppressing their proliferation, invasion, and metastasis and inducing cancer cell apoptosis. Consequently, AFP plays a crucial role in facilitating the malignant progression of HCC. Nevertheless, AFP is a double-edged sword in the development, diagnosis, and treatment of HCC (**Figure 5**). For clinical practice, measuring AFP levels holds immense significance for the early detection, prognosis, and treatment of HCC. By monitoring the fluctuations in AFP levels, medical professionals can monitor disease progression and treatment response, guiding clinical decision-making. Furthermore, clinicians can use AFP vaccines to generate AFP-specific CD8^+^ T cells and kill cancer cells. In addition, AFP combined with immunotherapy, can improve the therapeutic efficacy.

In summary, AFP plays a significant role in the TME of HCC. It modulates the function of tumor-associated macrophages, increases the population of tumor stem cells, and facilitates the immune evasion of liver cancer cells. Further studies are needed to gain a comprehensive understanding of the underlying mechanisms through which AFP is involved in the TME of HCC and provide new approaches for treatment and prognosis prediction in HCC.

## Author contributions

YL: Data curation, Resources, Supervision, Writing – original draft, Writing – review & editing. BL: Supervision, Writing – original draft, Writing – review & editing. ML: Funding acquisition, Investigation, Supervision, Writing – review & editing.
